# DRP-1, ezrin and E-cadherin expression and the association with esophageal squamous cell carcinoma

**DOI:** 10.3892/ol.2014.2114

**Published:** 2014-05-07

**Authors:** JIANWEN ZHAI, YANCHEN WANG, FUSHEN YANG, JIGANG HU, QINGBIN QI, YANLI ZHANG

**Affiliations:** Department of Cardiothoracic Surgery, The Affiliated Hospital of Hebei University of Engineering, Handan, Hebei 056002, P.R. China

**Keywords:** DAPK, ezrin, E-cadherin, esophageal squamous cell carcinoma, immunohistochemistry, *in situ* hybridization

## Abstract

It has been shown that death-associated protein kinase (DAPK) family and E-cadherin play significant roles in the promotion of apoptosis and the suppression of cell adhesion and migration, and are involved in tumor metastasis. Ezrin, a cytoplasmic peripheral membrane protein, has been shown to interact with E-cadherin to participate in the metastasis of tumor cells. The present study aimed to investigate the expression of DRP-1 (a member of the DAPK family), ezrin and E-cadherin in esophageal squamous cell carcinoma (ESCC), and to analyze their association with clinicopathological factors in order to explore their potential in ESCC diagnosis. The expression of these genes was studied in tissue microarrays using *in situ* hybridization and immunohistochemistry methods in 76 specimens of ESCC and their paracancerous normal squamous epithelium tissues. Expression was statistically analyzed with regard to clinicopathological factors using χ^2^ and non-parametric tests. The expression level of DRP-1 was significantly different between the ESCC and paracancerous tissues (P<0.05). The expression level was correlated with the depth of invasion and lymph node metastasis (P<0.05). Abnormal E-cadherin expression was found to be associated with a high degree of cancer differentiation and lymph node metastasis (P<0.05). A positive correlation was observed between the expression of DRP-1 and E-cadherin (P<0.05). The expression of ezrin was found to be correlated with the depth of ESCC invasion, the degree of differentiation and lymph node metastasis (P<0.05). The high expression of ezrin has been previously shown to be correlated with the low or absent expression of E-cadherin. In conclusion, in ESCC, the expression levels of DRP-1, ezrin and E-cadherin were all reduced, and this reduction or absence of expression may have been attributed to ESCC tumorigenesis and progression. Simultaneous analyses of DRP-1, ezrin and E-cadherin expression levels would be useful to determine the malignancy and metastatic potential of ESCC, and these genes are consequently of potential use as biomarkers for the diagnosis and prognosis assessment of early-stage ESCC.

## Introduction

Esophageal squamous cell carcinoma (ESCC) is a common gastrointestinal cancer with a poor prognosis mainly due to metastasis. The death-associated protein kinase (DAPK) family, a family of pro-apoptotic proteins, has previously been discovered as one of the genes isolated by the ‘technical knock-out’ (TKO) approach in a functional screen, based on the random knockdown of gene expression (1). DAPK has been shown to be involved in a number of apoptotic signal transduction pathways, initiating tumorigenesis though unbalanced cell proliferation and death, though the accumulation of mutated genes and via a prolonged cell growth period (2–4). Ezrin has been demonstrated to be significant in metastasis (5). DAPK and ezrin interact with E-cadherin in metastasis. In the present study, the expression of DRP-1 (a member of the DAPK family), ezrin and E-cadherin was examined in tumor tissues excised from ESCC patients from Handan, Hebei, a high ESCC incidence area, using *in situ* hybridization and immunohistochemistry methods, and analyzed their roles in the carcinogenesis and development of ESCC in order to discover and develop novel biomarkers for the condition.

## Materials and methods

### Specimens and Patients

Surgical tissues were collected from 76 patients who underwent surgery in the Department of Thoracic Surgery of the Affiliated Hospital of Hebei University of Engineering (Handan, Hebei, China) between July 2008 and July 2010, and used to prepare tissue arrays. The patients included 49 males and 27 females (with a male to female ratio of 1.8:1), aged 39 to 73 years, with a median age of 61±5.1 years. None of the patients had received radiotherapy or chemotherapy prior to the surgery. In total, 26, 33 and 17 were confirmed to have well-, moderately- and poorly-differentiated ESCC by post-operative pathological examination, respectively. A total of 10 patients were stage T1, 21 were T2, 29 were T3 and 16 were T4. Overall, 41 patients presented with lymphatic metastasis, while 35 did not. The samples were provided by the pathological department of the Affiliated Hospital of Hebei University of Engineering (Handan, China). This study was approved by the ethics committee of the Affiliated Hospital of Hebei University of Engineering.

### Methods

#### Tissue Array

Tissue paraffin blocks were sectioned and stained with hematoxylin and eosin (HE). To prepare the tissue arrays (10×8 mm), eight holes (2 mm in diameter) were created using Beecher Tissue Arrayer (Beecher Instruments, Inc., Sun Prairie, WI, USA) on a control block, and filled up with tissues obtained from the donor blocks according to positions precisely mapped on HE film. The identification number of the tissue was recorded for each hole. For each sample, tumor and paracancerous normal (3–5 cm away from the tumor tissue) tissues were used. The arrays were sectioned to a thickness of 3–4 μm, melted, stained with HE and examined by pathologists prior to subsequent analysis.

### Immunohistochemistry and in situ hybridization

The tissue sections were analyzed immunohistochemically using an immunohistochemistry kit (Beijing Zhongshan Jinqiao Biological Technology, Ltd., Beijing, China), following the manufacturer’s instructions. Rabbit antihuman polyclonal DAPK anybody (Boster, Wuhan, China) was diluted 150 times prior to use and counterstained with diaminobenzidine (Zymed Co., Invitrogen Life Technologies, Carlsbad, CA, USA), according to manufacturer’s instructions. For the control, phosphate-buffered saline was used in place of the primary antibody. All solutions used in the *in situ* hybridization, such as streptavidin-biotin alkaline phosphatase system (SA-Bio-AP), 5-bromo-4-chloro-3-indolyl-phosphate (BCIP)/nitroblue tetrazolium (NBT), were purchased from Wuhan Boster Biological Technology, Ltd. (Wuhan, Hubei, China). 5′-biotin-labeled, phosphorothioated probe (5′-CAGCTCGCCACCTGCAACGA) was synthesized by Beijing Bioko Biotechnology (Beijing, China).

The specimens were dewaxed in freshly prepared xylene and hydrated in a series of ethanol solution, and then dipped in freshly prepared H_2_O_2_ solution (0.5%) for 30 min to deactivate any endogenous peroxidase. The slices were then immersed in freshly prepared 3% citrate buffer (pH 6.0) containing trypsin (0.01 g/l) and incubated at 37°C for 10 min to digest DNA binding proteins. Each slide was administered 20 μl pre-heated (42°C) pre-hybridization solution without the probe and incubated for 4 h. The slide was then administered the hybridization solution with the probe (1 ng/l), and hybridized in a moist chamber for 12 h at 42°C. Subsequent to being washed with 0.1× standard sodium citrate at 42°C, SA-Bio-AP was added and the slides were incubated at 37°C for 10 min. The slides were then washed and administered BCIP/NBT for coloration in the dark for 2 to 4 h. The negative control was treated as the samples, but without the probe.

### Evaluation criteria

DRP-1 staining was considered positive if light-yellow to brown-colored granules were seen inside the cytoplasm. The expression level was graded based on the percentage of positive cells and the staining intensity, as previously described (6) and as follows: Samples with <10% positive cells scored 1, 10–50% scored 2 and >50% scored 3. Based on staining intensity, negative staining scored 0, light yellow (or blue) scored 1, medium light yellow (or blue) scored 2 and brown-yellow (or purple-blue) scored 3. Categorically, score 0 was graded as (−), 2 as (+) and ≥3 as (++), where (++) represented normal expression, (−) no expression and (+) reduced expression.

For ezrin staining, the cells with brown granules distributed diffusely in the cytoplasm were rated as positive. Based on the staining intensity and the number of positive cells, the ezrin expression was classified into (−) no expression, (+) with 50% of cells positive or with light staining, and (++) with ≥50% of cells being positive and highly stained (7), where (++) represents overexpression.

For E-cadherin expression, the cells with small yellow or brown granules on the membrane were considered to be positive, while those showing granules in the cytoplasm, but not on membrane, were rated negative. The expression level of E-cadherin was graded according to Gonzalez’s criteria, where (−) represents cells with no staining, (+) with <75% of cells stained and (++) with ≥75% cells being positive (8). Expression at (−) to (+) was considered to be negative or reduced, or abnormal expression.

### Statistical analysis

The data were analyzed with the aid of SPSS 13.0 software (SPSS, Inc., Chicago, IL, USA). The differences were tested by χ^2^ test, and Spearman’s rank correlation was performed. P<0.05 was considered to indicate a significant difference.

## Results

### Expression of DRP-1, ezrin and E-cadherin in ESCC and paracancerous cells

**Figure 1:**
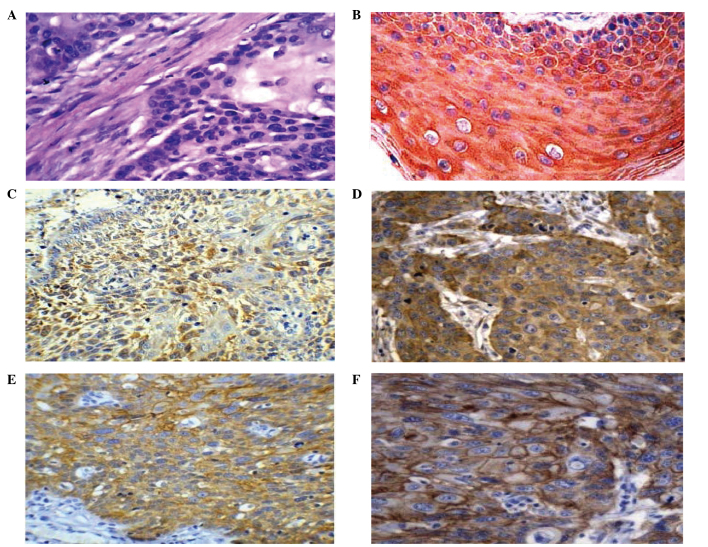
Streptavidin-peroxidase-biotin detection of DRP-1, ezrin and E-cadherin in ESCC and paracancerous tissues. (A) Expression of DRP-1 in the stratum spinosum and stratum corneum of the paracancerous tissues (x400). (B) Expression of DRP-1 in the cytoplasm of the cancer cells (x400). In our previous study, expression of ezrin was observed (C) in the stratum spinosum and stratum corneum of the paracancerous tissues and (D) in the cytoplasm of the cancer cells (x400). Expression of E-cadherin was observed (E) in the stratum spinosum and horny layer of the paracancerous tissues and (F) in the center of the cancer cells (x400) (17). ESCC, esophageal squamous cell carcinoma.

Following immunological staining, DRP-1 protein was localized in the cytoplasm, visualized as light yellow to brown granules in ESCC, but not in the control samples (Fig. 1A and B). Meanwhile, ezrin was observed to be expressed in the ESCC and paracancerous normal squamous epithelial cells, macrophages and lymphocytes in the interstitial tissues. In the epithelial tissue, ezrin was highly expressed in prickle cells and coenocytes, and weakly expressed or absent in basal cells. In the cancer tissues, ezrin was mainly expressed in the cytoplasm adjacent to the cell membrane, with few observed on the cell membrane (Fig. 1C and D). E-cadherin was mainly expressed on the cell membrane as brown granules in the cancer and paracancerous normal squamous epithelial tissues. In the epithelial tissues, it was intensely expressed in the basal and prickle cells (Fig. 1E and F). The expression levels of the three genes in the ESCC and paracancerous normal epithelial tissues were statistically different (Table I).

### Expression of DRP-1, ezrin and E-cadherin, and the clinical biology of ESCC

The present analysis indicated that the expression of DRP-1 was not associated with the age or gender of the patients, nor with the degree of tumor differentiation, but that it was associated with the invasiveness of the tumor and lymph node metastasis. Ezrin expression was associated with the tumor invasiveness and lymph node metastasis, but not with patient age and gender, or tumor size and degree of differentiation. Meanwhile, no association was found between the expression of E-cadherin and patient age and gender, or tumor size and invasiveness, however, an association with tumor differentiation and lymph node metastasis was observed (Table II).

### Associations among the expression of DRP-1, ezrin and E-cadherin in ESCC

In the 76 ESCC samples, 27 were positive and 49 were negative for DRP-1. Of the positive and negative samples, 15 were positive and 6 were negative for E-cadherin, respectively. Therefore, there was a higher percentage of samples with E-cadherin expression in the DRP-1-positive samples than in the DRP-1-negative samples (P<0.05), indicating that the expression of the two genes was positively correlated. There were 22 and 47 ezrin-positive samples in the DRP-1-positive and -negative samples, respectively, indicating that there were significantly less ezrin samples in the DRP-1-positive samples than in the negative samples (P<0.05), indicating that the expression of the two genes was negatively correlated (Table III). In our previous study, a total of 16 and 43 positive samples were observed in the samples expressing normal or abnormal levels of E-cadherin, respectively, indicating that there was a decreased number of ezrin-positive samples in the tissues with normal E-cadherin expression than in those with abnormal expression, indicating that the two genes were negatively correlated (17).

## Discussion

Metastasis and recurrence are the basic characteristics of malignant tumors, with 90% of tumor patients succumbing to distant metastasis (9). Studies have shown that DRP-1 is a calcium calmodulin-regulated serine/threonine kinase involved in apoptotic and autophagic cell death, and the suppression of tumors and metastasis (10,11). Decreased or absent DRP-1 expression has been found in a number of tumor cells and tissues, which may be associated with the methylation changes at CpG sites, and play significant roles in tumorigenesis and development (12,13). Ezrin has been demonstrated to be involved in the interactions between the cells and between the cells and the stroma through regulating adhesion molecules and signal transduction. Ezrin therefore is significant in tumor progression, invasion and metastasis. A high level of ezrin expression has been shown to be positively associated with malignancy and metastasis, and has been observed to be an indicator of poor prognosis (14,15). The calcium dependent glycoprotein, E-cadherin, is widely distributed in epithelial cells. The main role of E-cadherin is to mediate cellular adhesion between homogenous cells, with functional roles in the cytoskeleton to maintain structural integrity and epithelial polarity. E-cadherin mediates the absence or decrease of cell adhesion, which is an important step in the metastasis of the majority of tumors. Cell adhesion is weakened by this decrease or absence of E-cadherin expression, resulting in tumor cells that are easy to separate and that can grow invasively, leading to metastasis (16). Therefore, the three genes, DRP-1, ezrin and E-cadherin, are of significance in tumorigenesis and disease progression.

Results from the present study showed that although DRP-1 was expressed in the ESCC tissues and paracancerous cells, the expression level was significantly lower in the ESCC cells, indicating that tumors with lower DRP-1 expression are highly invasive. Furthermore, the results showed that the abnormal expression of the DRP-1 gene was associated with the depth of the invasion of the cancer and lymph node metastasis, indicating that abnormal expression may be used as an indicator for a poor prognosis in order to improve our understanding of the biology of the cancer. The DRP-1 gene may also provide a novel option for the early diagnosis and treatment of ESCC. Ezrin was highly expressed to a significant degree in the ESCC tissues compared with the paracancerous cells, indicating that it may play roles in the progression and metastasis of ESCC. Furthermore, its expression was found to be associated with the invasiveness of the cancer and lymph node metastasis, indicating that the expression of ezrin is an indication of tumor invasion and lymph node metastasis in patients, thereby resulting in a poor prognosis. Therefore, the detection of ezrin expression can be used to determine an early prognosis (17). In ovarian carcinoma, weak or absent ezrin expression in serous ovarian carcinoma has been associated with an unfavorable prognosis in patients (18). In the present study, E-cadherin expression was associated with the differentiation and lymph node metastasis of ESCC, but had no association with the age and gender of the patient, or the tumor size and invasiveness. The positive rate of E-cadherin expression was lower in the cancer tissues of the patients with lymph node metastasis than in those without (P<0.05), and was lower in the poorly-differentiated cancer tissues than in the well-differentiated tissues (P<0.05), indicating that a reduction in the expression of E-cadherin may be attributed to the differentiation and lymph node metastasis of ESCC. E-cadherin was similarly expressed in the cancer tissues with and without adventitia invasion, indicating that E-cadherin is not responsible for ESCC invasiveness. Further analysis indicated that there were negative and positive associations between DRP-1 and ezrin (P<0.01), and DRP-1 and E-cadherin (P<0.01), respectively, and the expression of ezrin and E-cadherin was found to be negatively associated (P<0.01). The results indicated that the three genes are interconnected, interactive and correlate with each other in their roles in tumorigenesis, progression, invasion and metastasis.

Tumor invasion and metastasis are multistage, multistep and multifactor processes, affected by the characteristics of the tumor cells, the overall immune status of the host and the characteristics of the local tissues being metastasized. The present results showed that DRP-1, ezrin and E-cadherin are involved in the tumorigenesis and metastasis of ESCC. DRP-1 and E-cadherin were shown to be negatively associated with ezrin, indicating that they may be antagonistic to each other. Simultaneous analysis of the expression of the three genes would aid in the determination of the differentiation degree, the invasiveness and the potential for metastasis, as well as serving as novel indicators for prognosis. Future studies are required to further elucidate the associations among the three genes.

## Figures and Tables

**Table I:** Expression of DRP-1, ezrin and E-cadherin in ESCC and paracancerous tissues.

	DRP-1, n (%)			Ezrin, n (%)			E-cadherin, n (%)		
						
Tissue	(+)	(−)	P-value	(+)	(−)	P-value	(+)	(−)	P-value
Paracancerous normal tissue	65 (85.5)	11 (14.4)		35 (46.1)	41 (53.9)		74 (97.36)	2 (2.63)	
Cancer tissue	27 (35.5)	49 (64.4)	<0.001	69 (90.7)	7 (9.2)	<0.001	21 (27.63)	55 (72.36)	<0.001
χ^2^	39.762			51.05			78.849		

ESCC, esophageal squamous cell carcinoma.

**Table II:** Association between expression of DRP-1, ezrin, E-cadherin and clinical parameters of ESCC.

		DRP-1 level, n					Ezrin level, n					E-cadherin level, n				
										
Clinical parameters	n	−	+	++	Positive, %	P-value	−	+	++	Positive, %	P-value	−	+	++	Positive, %	P-value
Gender																
Male	49	21	10	18	36.7		9	19	21	42.9		25	10	14	28.6	
Female	27	11	7	9	33.3	0.838	4	10	13	48.1	0.621	17	3	7	25.9	0.702
z_c_			−0.205					−0.494					−0.383			
Age, years																
<60	44	17	11	16	36.4		10	15	19	43.2		27	4	13	29.5	
≥60	32	16	5	11	34.4	0.844	8	11	13	40.6	0.796	15	9	8	25.0	0.467
z_c_			−0.196					−0.259					−0.728			
Tumor size, cm																
<5	33	14	9	10	30.3		15	6	12	36.4		16	8	9	27.3	
≥5	43	18	8	17	39.5	0.635	19	5	19	44.2	0.681	26	5	12	27.9	0.736
z_c_			−0.475					−0.412					−0.338			
Differentiation																
Well	26	11	6	9	34.6		8	8	10	38.5		8	6	12	46.2	
Moderate	33	14	7	12	36.4		9	11	13	39.4		21	5	7	21.2	
Low	17	7	4	6	35.3	0.997	4	5	8	47.1	0.841	13	2	2	11.8	0.006
χ^2^ value			0.006					0.346					10.148			
Invasion																
Not to adventitia	31	22	4	5	16.1		14	10	7	22.6		16	5	10	32.3	
To tissue outside adventitia	45	10	13	22	48.9	0.000	10	13	22	48.9	0.012	26	8	11	24.4	0.450
z_c_			−3.981					−2.522					−0.775			
Lymph node metastasis																
Yes	41	13	8	20	48.8		10	10	21	51.2		28	7	6	14.6	
No	35	19	9	7	20.0	0.016	14	12	9	25.7	0.032	14	6	15	42.8	0.006
z_c_			−2.409					−2.141					−2.758			

ESCC, esophageal squamous cell carcinoma.

**Table III:** Association between expression of DRP-1, ezrin and E-cadherin in ESCC.

		Ezrin			E-cadherin		
					
Sample	n	(+)	(−)	P-value	(+)	(−)	P-value
DRP-1-positive	27	22	5		15	12	
DRP-1-negative	49	47	2	<0.05	6	43	<0.01
χ^2^ value		4.338			16.330		

ESCC, esophageal squamous cell carcinoma.
